# Sinonasal metastasis as the initial presentation of renal clear cell carcinoma: a dual-center retrospective study and proposed diagnostic algorithm

**DOI:** 10.3389/fonc.2026.1759254

**Published:** 2026-03-19

**Authors:** Kai Sun, Zhiyu Zhang, Hefeng Wang, Min Chen, Weihua Yan, Guanggang Shi, Xiaoting Wang, Yan Jiang, Wenhui Pang

**Affiliations:** 1 Department of Otolaryngology-Head and Neck Surgery, The Affiliated Hospital of Qingdao University, Qingdao, Shandong, China; 2 Department of Otolaryngology Head and Neck Surgery, Shandong Provincial Hospital Affiliated to Shandong First Medical University, Jinan, China; 3 Qilu Hospital (Qingdao), Cheeloo College of Medicine, Shandong University, Qingdao, Shandong, China; 4 Department of Pathology, The Affiliated Hospital of Qingdao University, Qingdao, Shandong, China

**Keywords:** clear cell renal cell carcinoma, diagnostic challenge, endoscopic surgery, oligometastasis, sinonasal metastasis

## Abstract

**Objective:**

To investigate the clinical characteristics, diagnostic approaches, and treatment strategies for metastatic clear cell renal cell carcinoma (ccRCC) initially presenting with sinonasal involvement, thereby enhancing early recognition and optimal management.

**Method:**

We conducted a retrospective dual-center cohort study on five patients with ccRCC who presented with initial nasal symptoms. These patients were admitted to the otolaryngology departments of two regional medical centers between August 2011 and September 2024. All patients underwent endoscopic sinus surgery, and a detailed review of the patients’ clinical presentations, imaging and histopathological findings, treatment regimens (endoscopic resection followed by adjuvant therapy), and oncologic outcomes.

**Result:**

All five patients were male, aged 55 to 71 years with a median age of 66 years. Primary symptoms included epistaxis (3/5), nasal obstruction (5/5), and decreased vision (1/5). The diagnosis was confirmed by preoperative biopsy in three cases and intraoperative pathological examination in the remaining two. Complete tumor resection was achieved via endoscopic nasal surgery. Over a follow-up ranging from 17 to 50 months, three patients were alive with no evidence of disease (ANED) at 40, 48 and 50 months, respectively; one patient with concurrent lung metastasis died at 17 months; and one patient was lost to follow-up at 36 months. The median follow-up was 38 months (range: 17–50).

**Conclusion:**

Our findings support the role of complete endoscopic resection combined with systemic therapy in oligometastatic ccRCC. The proposed diagnostic algorithm provides a structured pathway to mitigate diagnostic delay. Heightened clinical vigilance and the application of such a pathway are essential for early diagnosis and optimal management in patients presenting with unexplained sinonasal symptoms.

## 1 Introduction

Clear cell renal cell carcinoma is the most prevalent type of kidney cancer, accounting for approximately 85% to 90% of renal malignancies. It predominantly affects males aged 40 to 60 years and represents about 2% of global cancer deaths (1). Only 6% of patients experience metastasis to the head and neck region, with the tonsils, nasal cavity, and paranasal sinuses being common sites. Less than 1% of metastases occur in the paranasal sinuses and nasal cavity (2). According to statistics from the National Cancer Center of China, the annual incidence of renal cell carcinoma is about 3.5 per 100,000, with metastatic ccRCC accounting for 75%-80% of cases (2). Most patients with nasal cavity and paranasal sinus metastases have a history of renal cell carcinoma, and some develop metastases long after nephrectomy. However, cases presenting with sinonasal metastases as the initial manifestation are exceedingly rare and pose a significant diagnostic challenge (3). Metastatic ccRCC to the nasal cavity and paranasal sinuses is often misdiagnosed as a primary tumor, leading to delayed treatment. This study analyzes five such cases to elucidate their clinical characteristics, highlight diagnostic challenges, and evaluate treatment outcomes, thereby providing insights to improve early diagnosis and management.

## 2 Materials and methods

### 2.1 Case records

This dual-center retrospective study received approval from the Medical Ethics Committee of the Affiliated Hospital of Qingdao University and the Ethics Committee of Shandong Provincial Hospital. Written informed consent was provided by all participants. Five cases of metastatic renal clear cell carcinoma with nasal symptoms as initial manifestations were enrolled from two medical centers. These five cases represent all consecutive patients diagnosed with sinonasal metastasis as the initial presentation of ccRCC at the two participating centers during the study period (August 2011 to September 2024). During this period, a total of 1,247 patients with primary sinonasal malignancies were treated at both institutions. To ensure complete case capture, we cross-referenced our otolaryngology surgical databases with the pathology archives and oncology patient registries of both centers using the keywords “renal cell carcinoma,” “metastasis,” and “sinonasal/paranasal sinus.” No additional cases were identified through this cross-referencing process. These cases were systematically identified from the otolaryngology departments of two major regional tertiary referral centers, which serve a large population base and are likely to capture the majority of such rare presentations in the region. All patients were male with a median age of 66 years (range: 55–71 years). Nasal endoscopy or electronic fiber laryngoscopy was performed preoperatively, alongside CT and MRI scan of the paranasal sinus. Tumor locations included the nasal cavity, maxillary sinus, ethmoid sinus, sphenoid sinus, frontal sinus, and clivus, with tumors exhibiting smooth surfaces, reddish coloration, and easy bleeding upon palpation. Confirmation of diagnosis was obtained by preoperative biopsy in three cases and intraoperative pathological examination in the remaining two. Further examination revealed cervical lymph node metastasis in one patient, and both cervical lymph node and lung metastasis in another.

Inclusion Criteria: ①Pathologically confirmed nasal cavity and sinus metastasis from ccRCC; ②Initial symptoms in the nasal cavity and sinuses with no prior history of kidney ccRCC; ③No prior radiotherapy or chemotherapy before surgery; ④Complete clinical data.
Exclusion Criteria: Patients with concurrent malignant tumors or incomplete follow-up data were excluded.

### 2.2 Data collection and analysis

Clinical, imaging, and pathological data were extracted from electronic medical records using a standardized case report form. Descriptive statistics were applied for demographic and clinical variables. Continuous variables are presented as median (range), and categorical variables as counts (percentages).

### 2.3 Literature review

A non-systematic literature review was conducted to contextualize our findings. PubMed and CNKI databases were searched for relevant articles published between 2000 and 2024 using keywords including “renal cell carcinoma,” “sinonasal metastasis,” and “initial presentation.” Selected articles were reviewed narratively to compare clinical features and outcomes with our case series.

### 2.4 Imaging studies

Retrospective analysis of CT and MRI imaging features was conducted to observe the extent of lesion involvement and determine if there was invasion of the orbit, skull base, or other regions.

### 2.5 Laboratory tests

Laboratory tests included complete blood count, biochemical profiles, histopathology, and immunohistochemistry.

### 2.6 Treatment plan

Routine preoperative examinations were conducted upon admission, including control and treatment of underlying diseases. After exclusion of surgical contraindications, all five patients underwent transnasal endoscopic sinus surgery and tumor resection under general anesthesia. The sinus ostia were fully exposed to allow complete tumor excision and debridement of affected skull base bone and necrotic mucosa. Resected tissue was sent for pathological examination. Patients received postoperative radiotherapy or targeted therapy, with regular CT and MRI scans.

## 3 Results

A total of five male patients with sinonasal metastasis as the initial presentation of ccRCC were included in this dual-center analysis. The findings are presented below in four subsections.

### 3.1 Demographics and clinical presentation

All patients were male with a median age of 66 years (range: 55–71 years). The primary presenting symptoms were nasal obstruction (5/5, 100%), epistaxis (3/5, 60%), and decreased vision (1/5, 20%). Detailed clinical characteristics for each patient are summarized in **Table 1**.

**Table 1:** Clinical characteristics, treatment, and outcomes of five patients with sinonasal metastasis as the initial presentation of ccRCC.

Patient No.	Age	Gender	Principal symptoms	Tumor location	Other metastatic site at diagnosis	Diagnostic method	Adjuvant therapy	Follow-up (months)	Treatment response (RECIST v1.1)	Outcome
1	71	male	nasal obstruction accompanied by visual impairment	the sphenoid sinus and sellar region	no	intraoperative pathological examination	surgery and targeted therapy	48	CR	ANED
2	55	male	nasal obstruction with epistaxis	nasal cavity	no	preoperative biopsy	surgery and targeted therapy	50	PR	ANED
3	67	male	nasal obstruction	nasal cavity and ethmoid sinus	cervical lymph nodes	intraoperative pathological examination	surgery only	36	Not assessed	Not assessed
4	71	male	nasal obstruction with epistaxis	the nasal cavity, ethmoid sinus, and orbit	cervical lymph nodes and lung	preoperative biopsy	surgery and radiotherapy	17	PD	Death
5	66	male	nasal obstruction with epistaxis	nasal cavity and ethmoid sinus	no	preoperative biopsy	surgery and targeted therapy	40	CR	ANED

Follow-up was calculated from the date of sinonasal surgery. ANED, alive with no evidence of disease; PR, Partial Response; CR, Complete Response; PD, Progressive Disease; Not assessed, patient lost to follow-up before response could be evaluated.

### 3.2 Imaging findings

**Figure 1:**
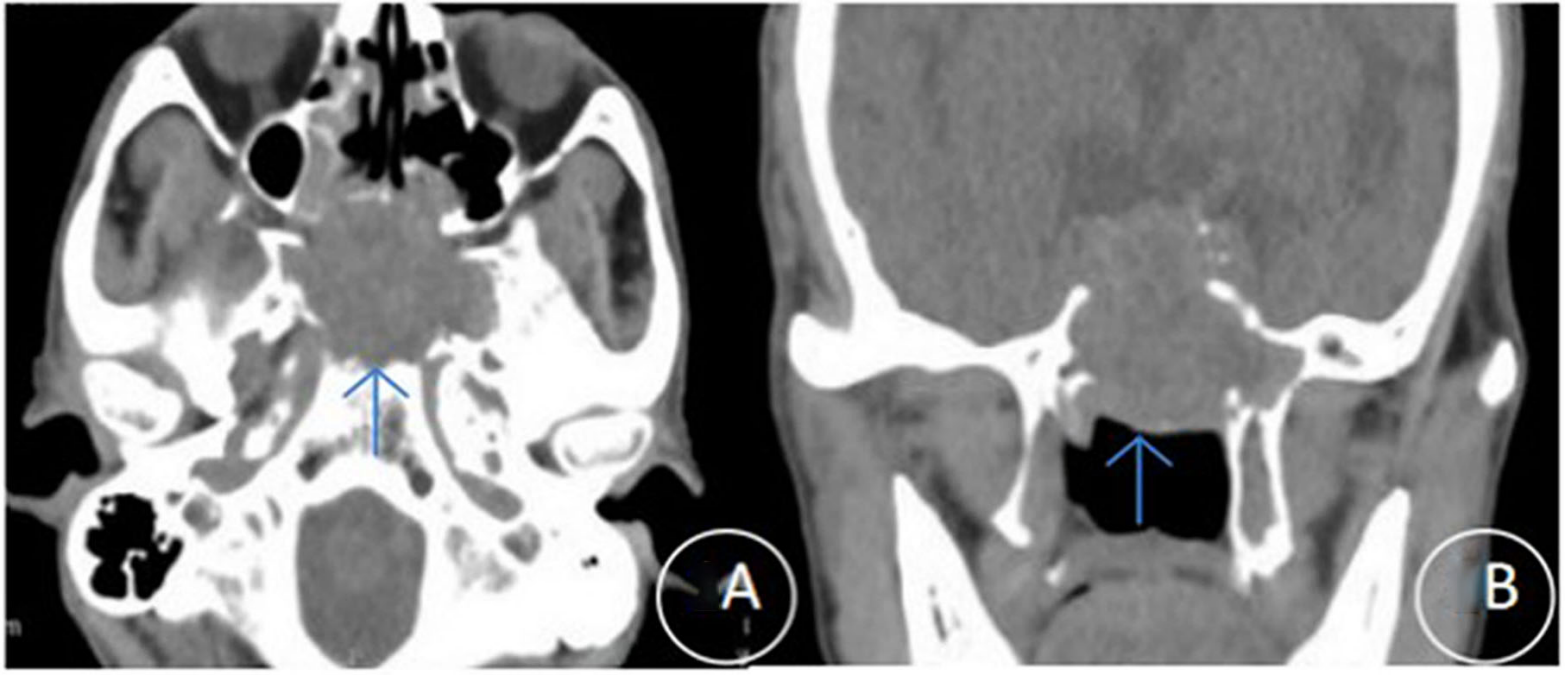
Preoperative CT characteristics of a representative sphenoid sinus tumor. **(A, B)** CT scans **(A)** Axial and **(B)** coronal views show a sphenoid sinus mass (arrows) with associated bone destruction of the clivus and skull base.

**Figure 2:**
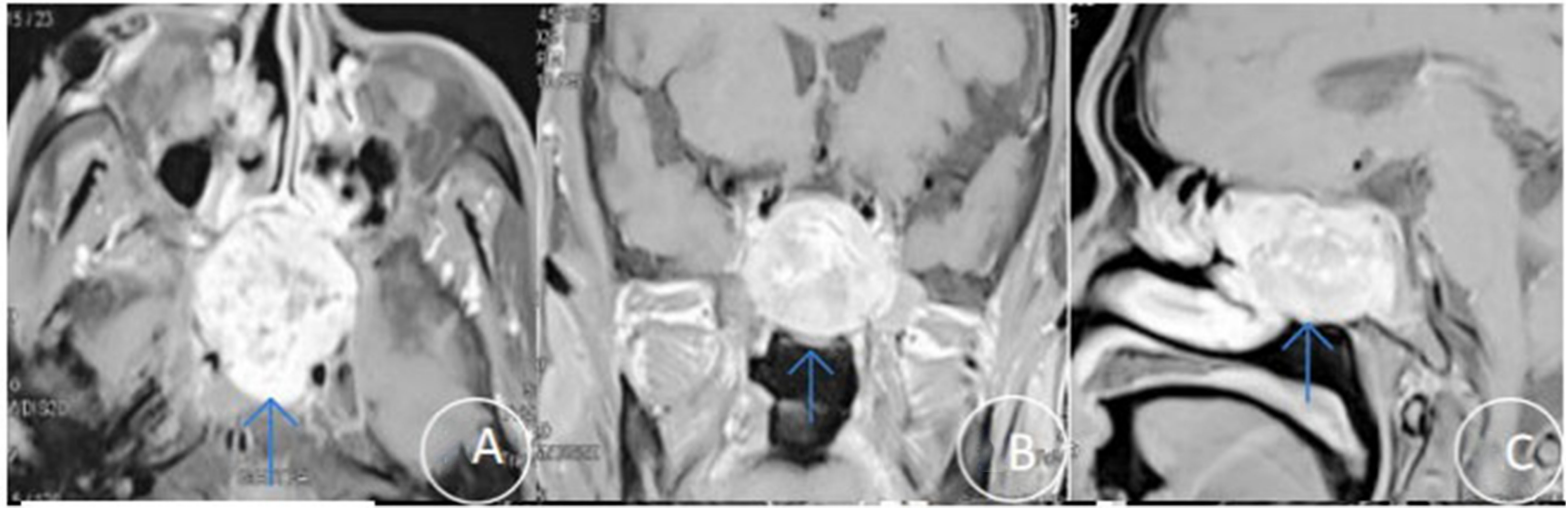
Preoperative MRI characteristics of a representative sphenoid sinus tumor. **(A–C)** Contrast-enhanced T1-weighted MRI **(A)** axial, **(B)** coronal, and **(C)** sagittal images delineate the tumor (arrows) invading the clivus, cavernous sinus, and protruding into the nasopharyngeal cavity.

Preoperative sinus CT scans revealed soft tissue masses with osteolytic bone destruction, most commonly involving the clivus and sphenoid sinus regions (**Figures 1A, B**). Contrast-enhanced MRI further delineated the tumors, showing occupying lesions in the anterior clinoid region and sphenoid sinus, with displacement of adjacent critical structures such as the pituitary gland and cavernous sinuses (**Figures 2A–C**). The imaging features were consistent with aggressive, hypervascular neoplasms.

### 3.3 Pathological and immunohistochemical features

**Figure 3:**
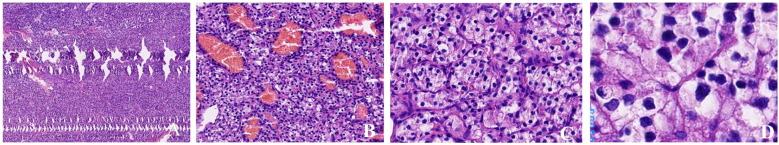
Histological characteristics of the tumor by H&E staining. **(A)** A well-demarcated metastatic nodule is present beneath the intact respiratory mucosa (×40). **(B)** The classic alveolar architecture, compartmentalized by a rich network of delicate, thin-walled blood vessels, is evident (×200). **(C)** The tumor exhibits a solid and nested growth pattern, identical to that seen in primary renal clear cell carcinomas (×400). **(D)** Tumor cells display characteristic cytologic features, including abundant clear cytoplasm and distinct cell borders, pathognomonic for clear cell renal cell carcinoma (×800).

**Figure 4:**
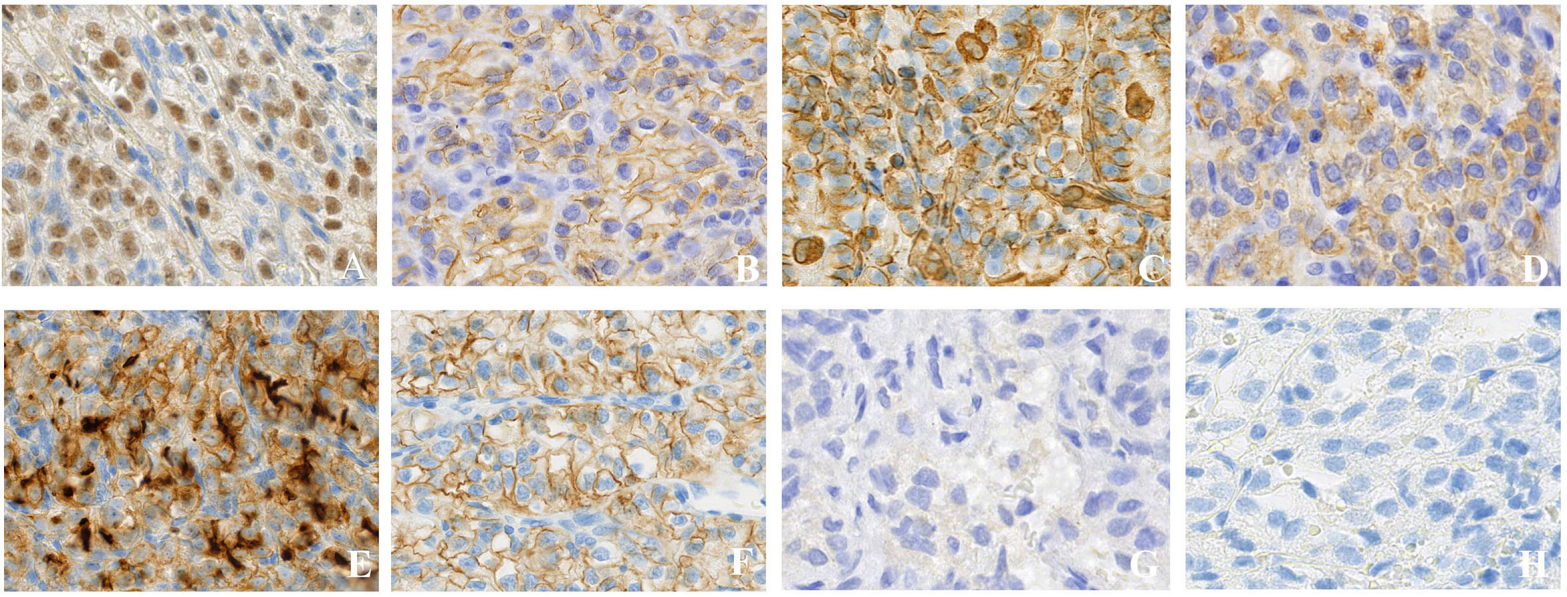
Immunohistochemical profile supports the diagnosis of clear cell renal cell carcinoma (ccRCC) (×400). **(A)** PAX-8: The tumor cells demonstrate strong nuclear staining, confirming renal tubular origin. **(B)** CA9: Diffuse and strong membranous reactivity, a hallmark of ccRCC. **(C)** Vimentin: Diffuse cytoplasmic immunoreactivity, characteristic of ccRCC. **(D)** CK (AE1/AE3): Tumor cells exhibit strong and diffuse membranous and cytoplasmic positivity. **(E)** CD10: Tumor cells are positive. **(F)** CD56: Tumor cells show membranous staining. **(G)** CK7: Tumor cells are negative, a feature arguing against chromophobe and papillary renal cell carcinomas. **(H)** Syn: Tumor cells are negative.

Histopathological examination of all resected specimens revealed tumors exhibiting an acinar or solid nested growth pattern, with a stroma rich in thin-walled blood vessels and areas of hemorrhage (**Figures 3A–D**). Tumor cells displayed characteristic abundant clear cytoplasm and round to irregular nuclei. Immunohistochemical analysis was pivotal for diagnosis, showing positivity for renal lineage markers PAX-8 (nuclear) and CA9 (membranous), as well as Vimentin, CK (AE1/AE3), CD10, and CD56 (**Figures 4A–F**). Staining was negative for CK7 (**Figure 4G**) and Syn (**Figure 4H**). The Ki-67 proliferative index was 30% across the cohort. This immunoprofile confirmed the diagnosis of metastatic clear cell renal cell carcinoma.

### 3.4 Treatment outcomes and survival analysis

All five patients underwent complete tumor resection via transnasal endoscopic surgery without major postoperative complications. The estimated intraoperative blood loss ranged from 50 mL to 200 mL (median: 100 mL). No patient required perioperative blood transfusion. Preoperative angioembolization was not performed in any case because: (1) none of the patients presented with active, uncontrollable epistaxis requiring emergency intervention; (2) all epistaxis episodes were self-limited or controlled with conservative measures prior to surgery; and (3) the surgical team (experienced in endoscopic skull base surgery) judged that complete resection with controlled hemostasis could be achieved using standard techniques, including controlled hypotension and bipolar electrocautery. This experience supports the individualized approach to embolization discussed in the manuscript. Treatment response was evaluated according to RECIST v1.1 criteria. Following surgery, patients received adjuvant therapy (targeted therapy or radiotherapy) as detailed in **Table 1**. The median follow-up duration was 38 months (range: 17–50 months). The 1-year progression-free survival rate was 100%. Three patients without evidence of distant metastasis at diagnosis remained alive with no evidence of disease (ANED) at 40, 48, and 50 months of follow-up, respectively. One patient with concurrent lung metastasis died at 17 months, and one patient with isolated lymph node metastasis was lost to follow-up at 36 months.

## 4 Discussion

Malignant tumors arising primarily in the nasal cavity and paranasal sinuses are far more common than metastases to this region. In comparison to other sites, metastatic tumors in these areas are exceedingly rare, most often originating from renal carcinoma, with clear cell renal carcinoma representing the highest proportion (4–6). Renal clear cell carcinoma is a malignant tumor that originates from the renal tubular epithelium and is the most common type of kidney cancer in adults. It has a metastasis rate of approximately 30% and a 5-year survival rate of about 50% (7, 8). Clear cell renal cell carcinoma often presents insidiously. Studies indicate that approximately one-third of patients have already developed metastases prior to the primary tumor diagnosis, while another third develop metastases during follow-up after diagnosis. There are also reported cases of metastasis occurring years after the initial radical nephrectomy (9). Renal clear cell carcinoma (ccRCC) is characterized by its proclivity for hematogenous metastasis. The Batson’s vertebral venous plexus, a valveless system connecting the pelvis to the cranium, provides the established anatomical rationale for its paradoxical spread to the head and neck region, bypassing the pulmonary filter (10, 11). The profound vascularity and hemorrhagic tendency observed intraoperatively in all our cases offer compelling clinical corroboration for this route of dissemination. This mechanism likely facilitated the delivery of tumor emboli directly to the vascular-rich sinonasal stroma, ultimately leading to the initial symptoms in our patients. Metastatic ccRCC to the sinonasal region typically exhibits a pattern of diffuse, osteolytic expansion rather than well-circumscribed growth. This locally aggressive behavior often results in significant destruction of the surrounding bony architecture, with potential for invasion into critical adjacent structures such as the orbit and skull base. Although the absolute number of cases is small, they were collected from high-volume tertiary care centers that serve as primary referral hubs for complex head and neck pathologies in their respective regions. This suggests that our series, while not exhaustive, likely reflects the clinical experience with this rare entity across a substantial patient population, thereby enhancing the generalizability of our observations regarding its presentation and management.

Accordingly, the clinical and imaging features of metastatic renal clear cell carcinoma in the nasal cavity and paranasal sinuses closely mimic those of the primary tumor. Common clinical symptoms include nasal obstruction and epistaxis, with some cases also reporting headaches or visual disturbances. Endoscopic examinations typically reveal smooth-surfaced, reddened, congested, and distended lesions that bleed easily upon touch (12). Consistent with previous literature, all our patients presented with non-specific symptoms such as epistaxis and nasal obstruction, leading to initial diagnostic challenges. Differentiating these lesions from other nasal cavity and paranasal sinus malignancies through imaging is also difficult. Non-contrast CT scans often show an irregularly shaped, heterogeneous density mass with poorly defined borders and destruction of surrounding tissues. Contrast-enhanced CT scans typically exhibit moderate to marked enhancement, often with a heterogeneous distribution (13). The profound vascularity of these metastases accounts for the common symptom of epistaxis and the significant intraoperative bleeding risk. MRI scans commonly show slightly low or low signal intensity on T1-weighted images, slightly high signal intensity on T2-weighted images, and partially heterogeneous signal intensity. After contrast enhancement, they display heterogeneous and marked enhancement (14). Preoperatively, these lesions are often misdiagnosed as primary tumors and are surgically removed. Only histopathological examination reveals them to be metastases from renal carcinoma. Misdiagnosis is particularly prevalent in patients without a history of renal carcinoma who initially present with symptoms involving the nasal cavity or paranasal sinuses. Therefore, for individuals without a history of renal carcinoma, multidisciplinary team (MDT) consultations involving radiology and pathology are crucial to avoid misdiagnosis.

Clinically, metastatic renal clear cell carcinoma in the nasal cavity and paranasal sinuses often needs to be differentiated from capillary hemangiomas, primary malignant tumors of the nasal cavity and paranasal sinuses, and metastatic tumors from other sites to the nasal cavity and paranasal sinuses (15–17). A comprehensive evaluation based on medical history, clinical presentation, histopathology, and immunohistochemical staining results is essential. Among these, histopathology combined with immunohistochemical staining is the most critical method for distinguishing metastatic renal clear cell carcinoma in the nasal cavity and paranasal sinuses. Histopathologically, tumor cells exhibit well-defined borders and nest-like arrangements. They display minimal atypia with abundant, clear cytoplasm and eosinophilic staining. Nuclei are round or oval with central positioning, occasionally containing nucleoli. The stroma shows hemorrhage, degeneration, necrosis, and minimal chronic inflammatory cell infiltration. Immunohistochemical staining demonstrating concurrent expression of cK (AEl/AE3), vimentin, PAX-8, and CD10 is diagnostically significant for renal clear cell carcinoma (18–20). PAX-8 expression is present in 98% of metastatic renal clear cell carcinomas. Studies indicate that CD10 serves as a key diagnostic criterion for distinguishing primary renal clear cell carcinoma of the nasal cavity and paranasal sinuses from metastatic disease, as metastatic renal clear cell carcinoma expresses CD10, whereas primary renal clear cell carcinoma of the nasal cavity and paranasal sinuses typically does not (21). Because CD10 is expressed in multiple normal tissues, it has high sensitivity but poor specificity. Therefore, combining antibodies such as Vimentin and PAX-8 is crucial for accurately diagnosing metastatic renal clear cell carcinoma (22). Furthermore, studies have shown that Ki-67, as a sensitive indicator of cell proliferation activity, exhibits positive expression suggesting higher tumor proliferation activity. Its expression level is positively correlated with tumor invasiveness and malignancy, and may be closely associated with prognosis (23). In our cases, the co-expression of PAX-8, CA9, and CD10, coupled with the negativity for markers like Syn, provided a definitive immunoprofile for metastatic ccRCC and effectively excluded other differential diagnoses. The elevated Ki-67 index (30%) in our series may reflect the aggressive biological behavior observed in these metastatic lesions.

Surgical Management and the Challenge of Hemorrhage Control. For patients with resectable oligometastatic disease, surgical excision remains the cornerstone of local control, a principle endorsed by the NCCN guidelines (24). The highly vascular nature of ccRCC metastases, however, presents a significant intraoperative challenge, as uncontrolled hemorrhage is a well-documented risk. Preoperative angioembolization is a well-established and effective adjunct to mitigate this risk, facilitate complete resection, and improve procedural safety (14). It is noteworthy, however, that in our case series, complete resection with controlled hemorrhage was successfully achieved without preoperative embolization. This was accomplished through meticulous endoscopic technique, including controlled hypotension and precise bipolar electrocautery. This experience underscores that the decision for embolization can be individualized, balancing factors such as tumor size, vascularity on imaging, and most critically, surgical expertise. Ultimately, the primary goal remains complete oncologic resection, for which both preoperative embolization and refined surgical technique are valuable tools in the armamentarium.

The prognosis of metastatic ccRCC is generally guarded, as it signifies systemic disease progression (25). This was evidenced in our series by the sole mortality case, which occurred in the patient with concurrent pulmonary metastasis at 17 months postoperatively. In contrast, the three patients without evidence of distant metastasis at diagnosis remained disease-free after combined modality treatment, with follow-up exceeding three years (40,48 and 50 months, respectively). This marked outcome disparity not only underscores the impact of tumor burden but also exemplifies the distinct clinical entity of “oligometastatic disease” (25). In the context of this study, oligometastatic disease was defined as the presence of ≤3 metastatic lesions confined to a single organ system (in this case, the sinonasal region) without evidence of widespread systemic dissemination (29). Patients with metastases involving multiple organs (e.g., sinonasal plus lung and/or bone) were classified as having polymetastatic disease. The oligometastatic state posits an intermediate stage of cancer spread with a limited number of metastases, wherein aggressive local ablation of all visible sites, in conjunction with systemic therapy, may potentially alter the natural history of the disease and improve survival (26–28). Our cohort serves as a compelling exemplar of this concept: the three long-term survivors presented with disease confined to the sinonasal region, fitting the paradigm of oligometastasis. Consequently, they achieved prolonged disease-free survival following a treatment strategy of complete metastasectomy via endoscopic surgery combined with adjuvant systemic treatment. Therefore, long-term survival is attainable in selected oligometastatic patients, as demonstrated in our series, through a multimodal strategy of complete surgical resection combined with systemic therapy such as the anti-angiogenic therapy sunitinib (27), despite the generally poor prognosis of metastatic ccRCC.

### 4.1 Mechanistic insights and clinical implications

**Figure 5:**
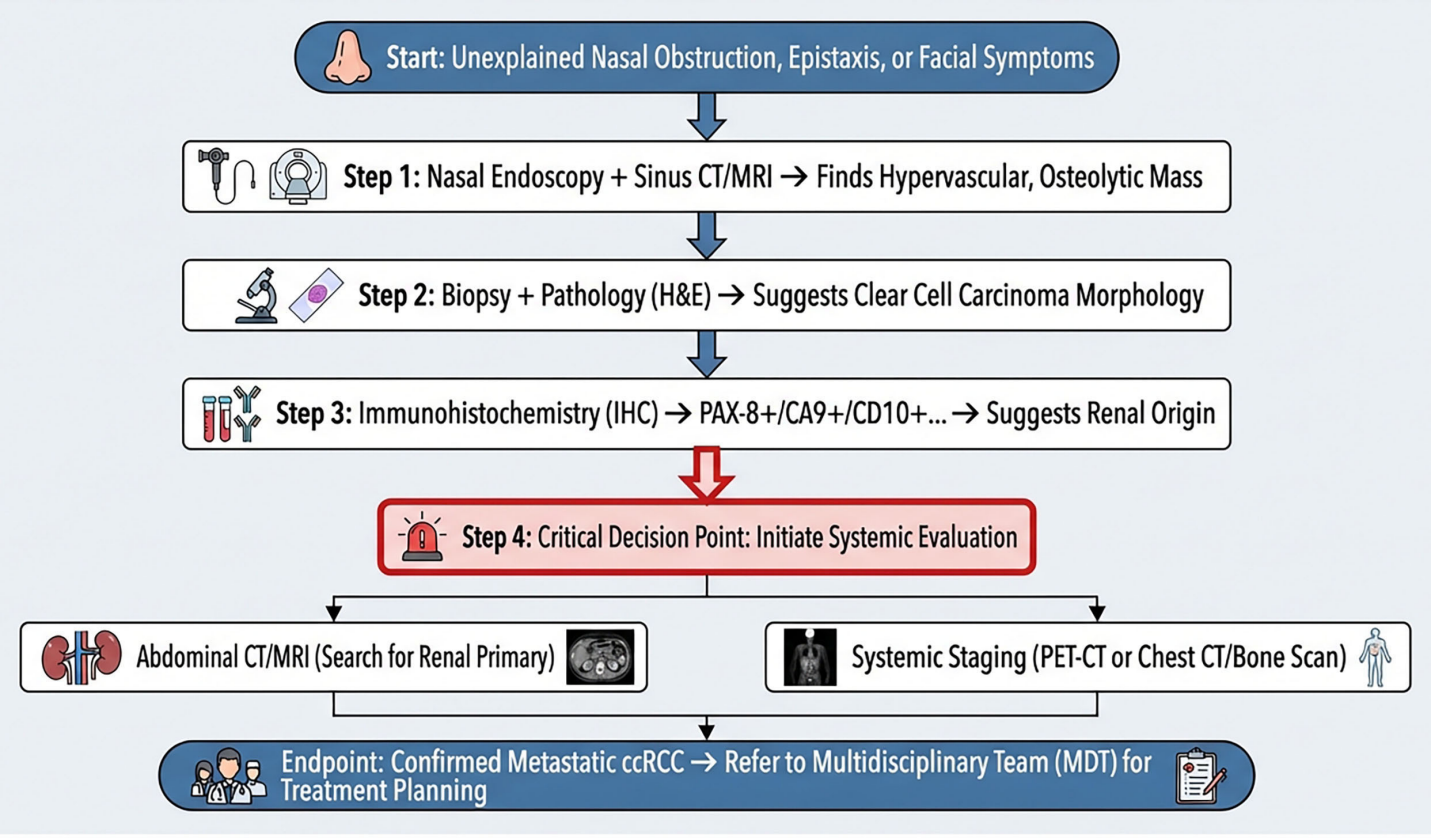
Proposed diagnostic algorithm for sinonasal masses suspicious for metastatic clear cell renal cell carcinoma. The algorithm integrates clinical presentation, imaging features, histopathological and immunohistochemical analysis, and mandatory renal evaluation with systemic staging, providing a structured pathway for early diagnosis and timely management.

The Batson’s plexus route not only provides an elegant anatomical explanation for this paradoxical metastatic pattern but also carries a direct and critical clinical imperative: any patient presenting with an unexplained, hypervascular sinonasal mass requires a systematic search for an occult primary tumor, with renal cell carcinoma at the top of the differential list. To translate this imperative into actionable clinical practice and to aid in the early recognition of this rare entity, we have synthesized our diagnostic experience into a stepwise diagnostic algorithm (**Figure 5**). This pragmatic framework integrates key findings from clinical presentation, endoscopic and cross-sectional imaging features, definitive histopathological and immunohistochemical analysis, and culminates in mandatory renal evaluation and systemic staging. Adherence to such a structured approach is vital to circumvent diagnostic delay and to ensure that patients receive appropriate, stage-directed therapy.

### 4.2 Limitations and future directions

The insights from this study must be interpreted in the context of its limitations. The small sample size, though reflective of the extreme rarity of the condition, inherently restricts the statistical power and generalizability of our conclusions. The retrospective design may introduce biases in data collection and patient selection. Furthermore, the small sample size and heterogeneity of adjuvant systemic therapy (sunitinib vs. radiotherapy vs. observation alone) preclude definitive conclusions regarding the comparative efficacy of different treatment regimens. Therefore, while our findings suggest a potential benefit of combined modality therapy in oligometastatic patients, these observations should be interpreted as hypothesis-generating rather than definitive evidence, and they require validation in larger, prospective cohorts. Future multicenter, prospective collaborative studies or registries are essential to accumulate larger cohorts, validate proposed diagnostic algorithms, rigorously compare treatment outcomes, and ultimately refine the management paradigm for this challenging clinical scenario.

## 5 Conclusion

In summary, this dual-center retrospective study demonstrates that sinonasal metastasis as the initial presentation of ccRCC, though rare, can be effectively managed with complete endoscopic resection combined with systemic therapy in oligometastatic patients. The proposed diagnostic algorithm provides a structured pathway to facilitate early recognition and timely intervention. Heightened clinical vigilance and multidisciplinary collaboration remain essential to avoid diagnostic delay and optimize outcomes in patients presenting with unexplained sinonasal symptoms.

## Data Availability

The original contributions presented in the study are included in the article/supplementary material. Further inquiries can be directed to the corresponding authors.
